# How can we breed for more water use-efficient sugarcane?

**DOI:** 10.1093/jxb/erw009

**Published:** 2016-02-02

**Authors:** Oula Ghannoum

**Affiliations:** ARC Centre of Excellence for Translational Photosynthesis, Hawkesbury Institute for the Environment, Western Sydney University, Penrith, NSW, Australia

**Keywords:** Conductance, crop productivity, germplasm, photosynthesis, sugarcane, transpiration efficiency, water-use efficiency

**Selection on the basis of physiological traits is hedged with obstacles in conventional breeding programmes – it is a little-explored concept. However, in this issue of *Journal of Experimental Botany* (pages 861–872), Jackson *et al.* present research in which the broad-sense heritability of leaf- and crop-level transpiration efficiency was tested within the framework of Australia’s main sugarcane breeding programme.**

Conventional breeding mostly consists of large-scale crosses followed by quick selection methods. To date, most breeding programmes do not use physiological indices, while some rely on experienced breeders walking through field or nursery trials and visually selecting the winners for the following stages. Further, breeders mostly select for vigour and disease resistance. Therefore, selecting for physiological traits, particularly something as complex as transpiration efficiency (TE), is deemed unworkable. The main obstacles include physiological traits often being complex, time-consuming to measure, subject to significant genotype–environment interactions, not clearly linked to genetic markers, and with their broad or narrow sense heritability weak or untested.

The key contribution of the study by Jackson *et al.* (2016) stems from the authors’ attempt to devise the least number of leaf gas exchange measurements required to infer statistically meaningful conclusions about variation and heritability in leaf TE, and the link with plant TE and yield in sugarcane. The main findings were significant genetic variations in plant TE and intrinsic leaf TE as measured by leaf intercellular CO_2_ concentration (C_i_); high broad-sense heritability for mean C_i_ (0.81); and C_i_ having a strong genetic correlation (–0.92) with plant TE at mid-range stomatal conductance (g_s_).

## Physiological definitions and variations of leaf transpiration efficiency

According to Fick’s law,

$$A=g_{s\_CO2}(C_{a}-C_{i})\;and\;E=g_{s\_H2O}(e_{i}-e_{a})$$ (1)

where *A* and *E* are the rates of leaf CO_2_ assimilation and transpiration (H_2_O), C_i_ and C_a_ are the leaf intercellular and ambient CO_2_ partial pressures, and e_i_ and e_a_ are the water vapour pressures inside the leaf and in the surrounding air, respectively. In addition, $g_{s\_H2O}=1.6g_{s\_CO2}$ , where $g_{s\_CO2}$ and $g_{s\_H2O}$ refer to the stomatal conductance for CO_2_ and water vapour, respectively; and 1.6 is the ratio of binary diffusivity of water vapour to that of CO_2_ in air (Farquhar *et al.*, 1989).

Accordingly, leaf-level TE (TE_L_) is given by:

$$TE_{L}=\frac{A}{E}=\frac{C_{a}\left(1-C_{i}/C_{a}\right)}{1.6\left(e_{i}-e_{a}\right)}$$ (2)

Assimilation rates depend on both g_s_ and photosynthetic biochemistry, while transpiration rates depend on boundary layer conductance, g_s_ and the leaf-to-air vapour pressure difference, which in turn depends on leaf temperature and the relative humidity of the surrounding air. Hence, this expression of TE is not ideal in screening for genetic differences because it is highly dependent on environmental conditions. A better expression that reflects a genotype-level trait is intrinsic TE (TE_i_), given by:

$$TE_{i}=\frac{A}{g_{s}}=1-\frac{C_{i}}{C_{a}}$$ (3)

**Fig. 1.**
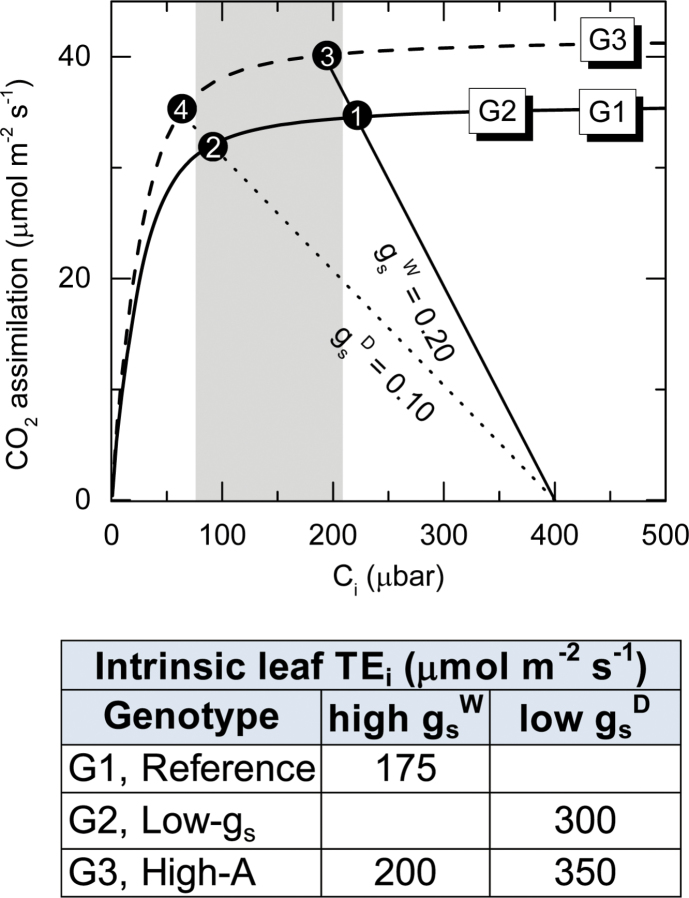
Modelled responses of C_4_ photosynthesis to changes in C_i_ at 25^o^C and saturating irradiance according to von Caemmerer *et al.* (2000). The modelling depicts three hypothetical genotypes. G1 (continuous line) and G3 (broken line) possess different photosynthetic capacity. G2 (continuous line) has similar photosynthetic capacity to G1, but operates with lower g_s_. Within each scenario, reduced g_s_ (due to low-g_s_ genotype or dry soil or air) increases TE_i_ at the expense of reduced *A*. Accordingly, TE_i_ increases by 73% while *A* decreases by 13%, when moving from points 2 to 1 (filled circles) in G1 and from 3 to 4 (filled circles) in G3; g_s_ decreases by 50%. Greater photosynthetic capacity in G3, relative to G1 and G2 genotypes, leads to increased TE_i_ at any given g_s_. G3 is the desirable genotype because it can potentially fix more CO_2_ in wet (e.g. high g_s_^W^) or dry (e.g. low g_s_^D^) conditions. The shaded area represents the ideal (C_i_) conditions under which genotypic screening gives the best population estimates of TE_i_ according to Jackson *et al.* (2016). At higher C_i_, *A* is no longer sensitive to changes in g_s_. At lower C_i_, *A* is highly sensitive to C_i_ giving erroneous estimates of TE_i_; or reduced g_s_ may be due to water stress, in which case C_i_ rises due to photosynthetic inhibition (Ghannoum, 2009), rendering TE_i_ estimates unreliable.

Reduced g_s_ leads to lower C_i_ and C_i_/C_a_, which represents an integrative parameter of TE_i_, reflecting changes in both *A* and g_s_ (equation 3). The contrasting influence of improved photosynthesis and reduced stomatal conductance on TE_i_ is illustrated in Fig. 1.

## Paradoxical relationship between crop yield and transpiration efficiency

Most rain-fed crops experience periods of water stress during the growing season. Hence, traits related to water use are critical for crop productivity and survival. Whole plant TE (TE_P_), the ratio of biomass produced to water used, is an important determinant of crop yield (Passioura, 1977), and crop yield (Y) can be expressed as:

$$Y=TE_{P}\times Water\;use\times Harvest\;index$$ (4)

**Fig. 2.**
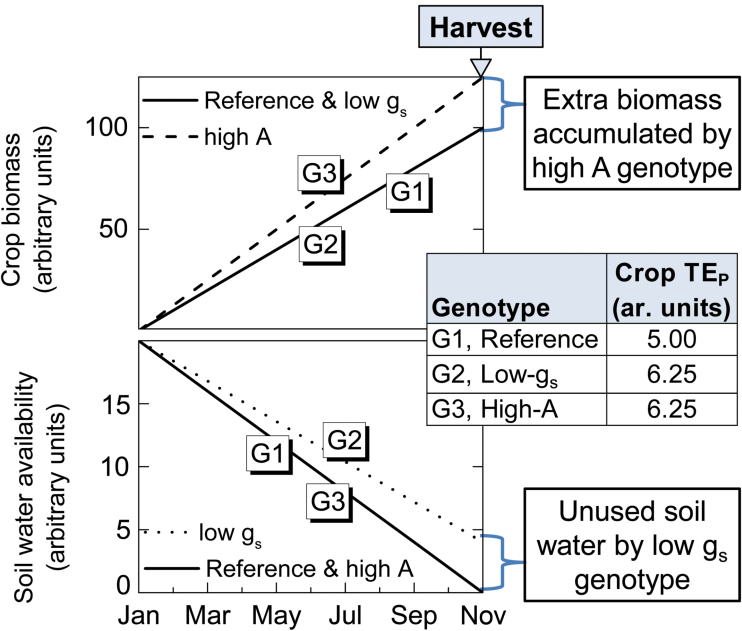
Illustration of sugarcane biomass accumulation and soil water availability (or use) between transplanting and harvest dates in three hypothetical genotypes. Within each scenario, reduced g_s_ (due to low-g_s_ genotype or dry soil or air) potentially increases TE_P_ without contributing to increased biomass accumulation at harvest relative to the reference genotype, G1. Most inefficiently for both rain-fed or irrigated crops, low-g_s_ genotypes or conditions imply that crops reach maturity without exhausting all available soil water at harvest, which translates into lower farm-level TE and productivity. Greater photosynthetic capacity in G3 relative to G1 and G2 genotypes potentially leads to increased TE_P_ and crop productivity at any stage during crop growth, including at harvest. G3 is the desirable genotype because it theoretically leads to greater productivity and TE_P_ in wet (high g_s_) or dry (low g_s_) conditions. In addition, the G3 genotype consumes all available soil water by harvest.

Greater TE_P_ may potentially lead to greater crop yield only if improved TE_P_ does not entail reduced water use. This is the case when improved TE_P_ results from improved *A* rather than reduced g_s_. These contrasting scenarios are illustrated in Fig. 2.

Sugarcane is a largely biomass crop, where harvest index is a fixed proportion of final biomass at harvest. This is not the case for grain crops, where traits and environmental conditions regulating the time of flowering and grain filling complicate the relationship between TE_P_, water use and crop yield. For example, grain crops that flower early may not have built enough biomass to fill lots of grains, while late-flowering crops may have too little water left in the soil during grain filling (Passioura, 2002). Hence, sugarcane is a crop where improved photosynthetic capacity will probably lead to greater potential crop yield.

## Perspectives

For most crops, and particularly for biomass crops such as sugarcane, improved TE is a desirable trait as long as it does not compromise total crop water use, which ultimately drives crop productivity in water-limited environments. Water-use is determined by a myriad of traits, including TE, root architecture, biomass partitioning and tissue respiration, amongst others (Farquhar *et al.*, 1989). Therefore, reporting good genetic correlations of leaf-level TE_i_ with plant TE and yield (Jackson *et al.*, 2016) is surprising, but good news for breeders and crop improvement.

Improved TE_i_ without compromising productivity is essentially a quest for improved photosynthetic capacity. Jackson *et al.* (2016) honed in on C_i_ as both an integrator of TE_i_ and a screening index, and have proposed that reduced C_i_ at any given stomatal conductance may result in improved yields in water-limited environments without compromising rates of crop water use and growth.

Finally, a word of caution. Given that atmospheric CO_2_ is rising and that C_a_ experienced by leaves in gas exchange cuvettes varies depending on photosynthetic capacity, amongst other factors, I suggest that C_i_/C_a_ is a more suitable screening index than C_i_ (equation 3). Selecting for lowered C_i_/C_a_ per stomatal conductance via breeding is highly desirable, especially for water-limited environments, and research should focus on developing low-cost, high-throughput screening tools that can be enticing for breeders.
