# How can we breed for more water use-efficient sugarcane?

**DOI:** 10.1093/jxb/erw126

**Published:** 2016-04-07

**Authors:** Oula Ghannoum

**Affiliations:** ARC Centre of Excellence for Translational Photosynthesis, Hawkesbury Institute for the Environment, Western Sydney University, Locked Bag, Penrith, NSW, Australia

Journal of Experimental Botany (2016) **67** (3): 557–559 doi:10.1093/jxb/erw009

In the above article incorrect units were given for intrinsic leaf TE_i_ in the table forming part of Fig. 1. A correct version of Fig. 1 is provided below.



